# High throughput RNAi screening identifies *ID1* as a synthetic sick/lethal gene interacting with the common *TP53* mutation R175H

**DOI:** 10.3892/or.2013.2953

**Published:** 2013-12-30

**Authors:** HIROO IMAI, SHUNSUKE KATO, YASUHIRO SAKAMOTO, YUICHI KAKUDO, HIDEKI SHIMODAIRA, CHIKASHI ISHIOKA

**Affiliations:** Department of Clinical Oncology, IDAC, Tohoku University, Sendai, Miyagi 980-8575, Japan

**Keywords:** *ID1*, *TP53* common mutation, synthetic sickness, gain of function

## Abstract

The *TP53* mutation (R175H) is one of the most common mutations in human cancer. It is a highly attractive strategy for cancer therapy to find the genes that lead the R175H-expressing cancer cells. The aim of this study was to identify the synthetic sick/lethal gene interacting with R175H. Using lentiviral bar-coded comprehensive shRNA library and a tetracycline-inducible R175H expressed in the SF126 human glioblastoma cell line (SF126-tet-R175H), we conducted high-throughput screening to identify the candidate genes that induce synthetic sickness/lethality in R175H-expressing cells. We identified 906 candidate gene suppressions that may lead to accelerated cell growth inhibition in the presence of R175H. Inhibitor of differentiation 1 (*ID1*) was one of the candidate genes, and its suppression by siRNA resulted in the acceleration of growth inhibition in cell lines both transiently and endogenously expressing R175H but not in *TP53*-null cell lines or other common p53 mutants (such as R273H). Flow cytometry analysis showed that *ID1* suppression resulted in G1 arrest, and the arrest was accelerated by the expression of R175H. *ID1* is a synthetic sick/lethal gene that interacts with R175H and is considered to be a novel molecular target for cancer therapy in R175H-expressing cells.

## Introduction

Synthetic sickness/lethality interaction is a highly attractive strategy for cancer therapy (1–4). For example, in cancer cells with a *KRAS* gene mutation, the inhibition of polo-like kinase 1 (PLK1) resulted in cell death (5). Similarly, cancer cells with the *KRAS* mutation were sensitive to the suppression of the serine/threonine kinase STK33 (6). Moreover, dysfunction of DNA double-strand break repair caused by mutations in *BRCA1* or *BRCA2* gene sensitized cells to the inhibition of poly-ADP ribose polymerase (PARP) enzymatic activity, resulting in chromosomal instability, cell cycle arrest, and subsequent apoptosis (7). This concept had been proved by a phase II trial where olaparib, a PARP inhibitor, provided objective antitumor activity in patients with a *BRCA1* or *BRCA2* mutation (8).

*TP53* is the most commonly mutated tumor suppressor gene in several different types of human cancer (9). *TP53* encodes the 393 amino acid p53 protein, which binds to specific DNA sequences in the regulatory region of downstream genes (10). A variety of cellular stressors including ultraviolet rays, ionizing radiation, chemotherapeutic drugs, and hypoxia stabilize the p53 protein, and post-translational modifications activate it; this results in various cellular responses including cell cycle arrest, DNA repair and apoptosis (11,12).

According to the *TP53* mutation databases, ~75% of the mutations are missense mutations (13,14); to date, >1,200 distinct missense mutations have been reported. Among them, those at residues Arg^175^(R175), Gly^245^(G245), Arg^248^(R248), Arg^249^(R249), Arg^273^(R273) and Arg^282^(R282) have been reported most frequently (15). The most common p53 mutant proteins caused by *TP53* hot-spot mutations are R175H, G245S, R248W, R248Q, R249S and R273H; these mutations cause a loss of the *trans*-activation function of downstream genes (16). However, some p53 mutants gain new functions that are not observed in wild-type p53 (so called gain-of-function mutations). For example, mice with the knock-in mutant p53 R172H and R270H, which correspond to human p53 R175H and R273H mutations, develop a variety of novel tumors such as lung adenocarcinoma, renal cancer, hepatocellular carcinoma, and intestinal carcinoma which are not generally observed in *TP53*-null mice (17). In addition, embryonic fibroblasts derived from p53 R172H knock-in mice gained activities of cell proliferation, DNA synthesis and retroviral transformation (18). Moreover, human p53 R273H or R248W interacted with Mre11 and suppressed the binding of the Mre11-Ras50-NBS1 (MRN) complex to DNA double-strand breaks, resulting in the chromosomal translocation and abrogation of the G2/M check point (19). According to these results, it has been hypothesized that some p53 mutant proteins, such as the activated K-ras protein, are oncogenic and contribute to carcinogenesis and cancer progression.

In the present study, we conducted high-throughput RNAi screening by a lentiviral gene suppression system to identify synthetic sick/lethal genes in the presence of p53 R175H, which accounts for ~6% of the missense mutations identified in human cancer (20). As a result, we identified that inhibitor of differentiation 1 (*ID1*) is the first gene that causes synthetic sickness when paired with p53 R175H mutant protein.

## Materials and methods

### Cell lines and culture

The stable SF126 cell line expressing the doxycycline (Dox)-inducible p53 R175H mutant (SF126-tet-R175H) was constructed according to the protocol described previously (21). In addition, SF126-tet-TON, which does not express p53, was used as a control (21). Mutant p53 was induced with 10 ng/ml doxycycline (Sigma-Aldrich, St. Louis, MO, USA). Five human cell lines including SKBr3 and HCC1395 (both derived from breast cancer), VMRC-LCD (derived from lung cancer), Detroit 562 (derived from head and neck cancer), and LS123 (derived from colon cancer) express p53 R175H endogenously. Colon cancer cell lines HT-29 and SW480 express p53 R273H endogenously. HCT116 (derived from colon cancer) expressed wild-type p53 endogenously. Four cell lines including PC3 (derived from prostate cancer), H1299 and Calu-1 (both derived from lung cancer), and SK-N-MC (derived from neuroblastomas) are *TP53*-null. PC3 was purchased from Cell Research Center for Biomedical Research, Institute of Development, Aging and Cancer, Tohoku University (Sendai, Japan). SKBr3, HCC1395, LS123, H1299, Calu-1, SK-N-MC, HCT116 and SW480 were purchased from American Type Culture Collection (ATCC; Manassas, VA, USA). Detroit 562 and VMRC-LCD were purchased from DS Farma Biomedical (Osaka, Japan) and Human Science Research Resources Bank (Tokyo, Japan), respectively. HT-29 was a gift from Dr John M. Mariadason. SKBr3, HCC1395, HT-29, SW480, H1299 and PC3 were cultured in RPMI-1640, and LS123, Calu-1, SK-N-MC, Detroit 562 and VMRC-LCD were cultured in minimum essential medium with 10% FBS at 37°C. The identity of SF126, PC3, HCC1395, LS123, H1299, Detroit 562, VMRC-LCD and HT-29 cells was tested using a set of 10 short tandem repeats produced by BEX Co., Ltd (Tokyo, Japan) in 2011. SKBr3, Calu-1, SK-N-MC, SW480, and HCT116 were passaged for <6 months.

### Mammalian p53 expression vectors

To express p53 R175H mutant, the p53 expression vectors pCR259-R175H and pCR259 were used (16,22). Each plasmid was transfected into *TP53*-null cells using Effectene Transfection Reagent (Qiagen, Hercules, CA, USA), following the manufacturer’s recommendations. Expression of p53 R175H was confirmed by western blot analysis.

### RNAi screening

One million SF126-tet-R175H and SF126-tet-TON cells were seeded in 10-cm culture plates for 24 h. The medium was removed from the plates and the Decode RNAi Viral Screening Library (Thermo Scientific Open Biosystems, Huntsville, AL, USA) was added to the plate at the multiplicity of infection (MOI) of 0.3 with serum-free medium. After 6 h, the medium was replaced with virus-free medium. After 48 h, puromycin was added at a final concentration of 2 mg/ml to select the infected cells. Finally, 7×10^6^ of lentivirus-infected SF126-tet-R175H and SF126-tet-TON cells were obtained. These cells theoretically contain 70,000 distinct shRNAs, and each cell should express a single shRNA product. These cells were divided into 2 groups, and each group was cultured with or without doxycycline for 10 days. Genomic DNA was extracted from each group using the Blood & Cell Culture DNA Mini kit (Qiagen), according to the manufacturer’s recommendation. Barcode sequences corresponding to specific shRNAs were amplified by the following primers located outside the barcode sequence: forward, 5′-caaggggctactttaggagcaattatcttg-3′ and reverse, 5′-ggttgattgttccagacgcgt-3′.

Amplified PCR products were separated in 1.5% TAE agarose gel and extracted using Wizard SV Gel and PCR Clean-Up system (Promega Corporation, Madison WI, USA). Each purified PCR product was labeled with Cy5 (doxycycline-on group) or Cy3 (doxycycline-off group) using Agilent’s Genomic DNA Labeling kit (Agilent Technologies, Inc., Santa Clara, CA, USA) and was hybridized on the barcode microarray in the hybridization oven at 65°C for 17 h. After hybridization, the arrays were scanned with the Agilent DNA microarray scanner to quantify log_2_ Cy5/Cy3. The ‘log2 Cy5/Cy3’ indicates increase and decrease of cells in the primary screening and negative value of ‘log2 Cy5/Cy3’ shows that the counts of R175H expressing cells (dyed with Cy5) is smaller than the counts of R175H unexpressed cells (dyed with Cy3). We conducted 2 independent experiments, and obtained 3 independent values of log2 Cy5/Cy3 were analyzed by Student’s t-test. Candidate genes were identified after analyzing raw data for each shRNA using the Gene Spring software (Agilent Technologies). Microarray data were deposited in GEO (accession no. 33362).

### Knockdown analysis of candidate genes using siRNA

The siRNAs of 50 candidate genes, identified from primary screening, were synthesized by Hokkaido System Science (Hokkaido, Japan). The sequences of synthesized siRNA for candidate genes are listed in Table I. *ID1-2* siRNA was synthesized as described previously (23). *TP53* siRNA was purchased from Applied Biosystems (Foster City, CA, USA), and *TP53-2* siRNA was purchased from Cell Signaling Technology, Inc. (Boston, MA, USA). A total of 3.5–5.0×10^3^ cells/well were seeded and incubated in a 96-well plate for 24 h. Each candidate siRNA and negative control siRNA was added to the cells to make a final concentration of 30 nM or 100 nM using DharmaFECT 1 (Dharmacon, Lafayette, CO, USA). Cell proliferation assays were performed using Cell Counting kit-8 (Dojin Laboratories, Kumamoto, Japan), as previously described (21).

### Western blot analysis

Cells were harvested and resuspended in lysis buffer containing 50 mM Tris-HCl (pH 8.0), 150 mM NaCl, 5 mM EDTA, and 1% protease inhibitor cocktail (Sigma-Aldrich). The lysate was subjected to western blot analysis, as previously described (24). Anti-p53 (Santa Cruz Biotechnology, Inc., Santa Cruz, CA, USA), anti-β-actin (Sigma-Aldrich), anti-Id1, and anti-GAPDH (Applied Biosystems) antibodies were used.

### Cell cycle analysis by FACS

A total of 1.5×10^4^ cells/plate were seeded and incubated in 6-cm culture plates for 24 h. The cells were further incubated in the presence of drugs for 48 h. These cells were collected, and FACS analysis was performed, as previously described (24).

## Results

### Screening of synthetic lethal genes that interact with p53 R175H mutant

**Figure 1:**
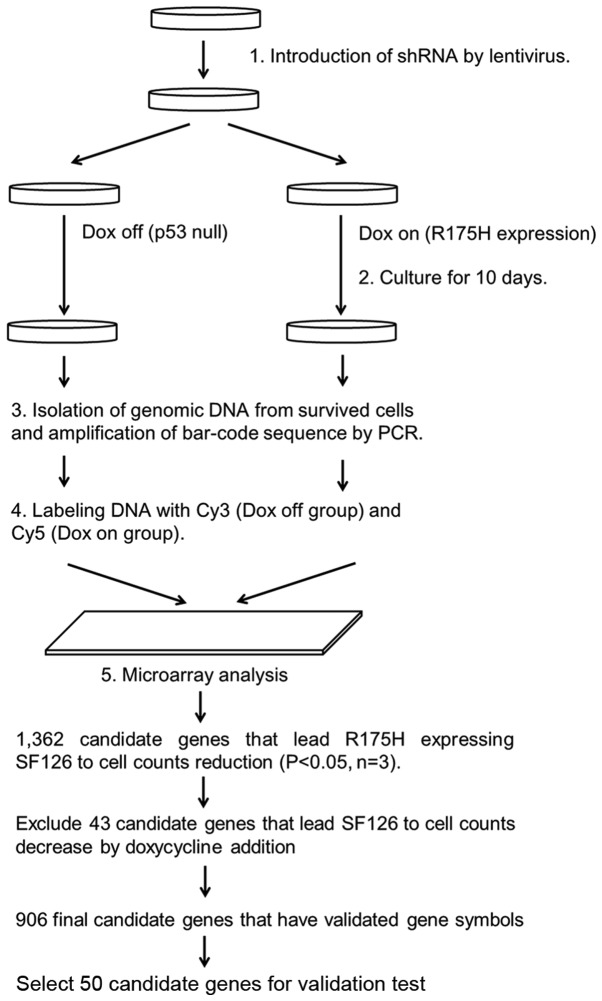
Flow chart of the comprehensive screening for synthetic lethal genes interacting with p53 R175H. Change in the abundance of a particular shRNA barcode is tracked for 10 days by competitive hybridization between the Dox-on and Dox-off groups. The cells in which shRNAs induced synthetic lethality with p53 R175H are selectively depleted from the Dox-on group. The same experiment was also conducted in SF126-tet-TON cells to exclude the possibility of doxycycline toxicity.

A flow chart of the high-throughput screening of synthetic lethal genes interacting with p53 R175H is shown in Fig. 1. By comparative analysis, 1,362 candidate genes were identified for synthetic lethality with p53 R175H expression in the SF126-tet-R175H cell line (p<0.05 according to t-test, n=3). Among these, 43 were excluded as suppression of these genes also resulted in decreasing cell numbers in SF126-tet-TON cells after doxycycline treatments (no R175H expression). In the remaining 1,319 genes, 906 genes have validated gene symbols, which have p-value <0.05. Among these, we selected 50 genes (21 genes from the group with the smallest p-values, 20 genes from the group with the largest fold-change, and 9 genes reproduced by different siRNA sequences) for further validation testing (Table I).

### Suppression of candidate genes by siRNA in p53 R175H expressing cell lines and TP53-null cell lines

To investigate whether the suppression of candidate genes by siRNA resulted in p53 R175H-dependent inhibition of cell growth, candidate gene siRNAs were transfected into cell lines expressing endogenous p53 R175H (SKBr3, LS123, HCC1395, Detroit 562 and VMRC-LCD) and *TP53*-null cells (PC3, H1299, SK-N-MC and Calu-1). We obtained the ratio of cell growth inhibition of candidate gene siRNA transfected cells for negative control siRNA transfected cells on day 4. In 50 candidate genes, suppression of *GYPC*, *NUP98, GP6, EFNA4* and *ID1* by siRNA significantly decreased the number of p53 R175H expressing cells compared with *TP53*-null cells (t-test) (Table II).

**Figure 2:**
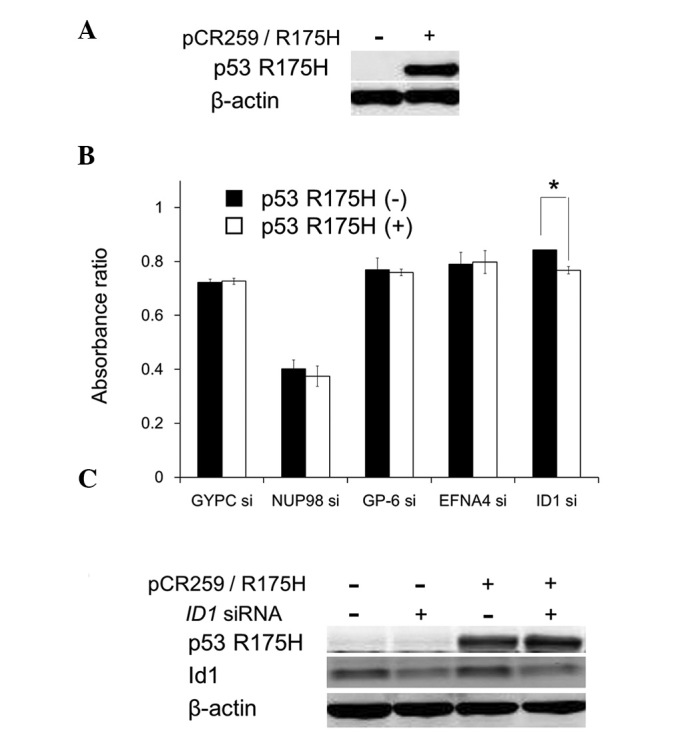
Suppression of candidate genes in PC3 cells with p53 R175H expression. Ninety-six hours after transfection of plasmid pCR259 or pCR259-R175H in PC3 cells, (A) p53 R175H expression was confirmed by western blotting, and (B) cell numbers were measured by performing cell proliferation assays. The vertical axis corresponds to the absorbance ratio of (candidate gene siRNA transfected cells)/(negative control siRNA transfected cells) for 5 candidate genes. Values shown are means ± SD (n=3). ^*^p<0.05 between p53 R175H null and p53 R175H expression. (C) Western blot analysis of Id1 and p53 R175H. Id1 expression was unchanged by R175H expression.

To examine whether the cell growth inhibition resulting from suppression of the candidate genes depends on p53 R175H expression, *GYPC, NUP98, GP6, EFNA4* and *ID1* were suppressed by specific siRNAs in PC3 cells transiently expressing p53 R175H. Suppression of these genes inhibited cell growth; however, among the candidate genes, *ID1* suppression significantly accelerated the cell growth inhibition under transient p53 R175H expression (Fig. 2A and B). *ID1* suppression and p53 R175H overexpression did not influence the other protein expression level (Fig. 2C). These results suggest that p53 R175H expression and *ID1* suppression cooperate to cause cell growth inhibition.

### Cell growth inhibition by ID1 and/or TP53 suppression in endogenously expressing p53 R175H, wt p53 cells and in TP53-null cells

**Figure 3:**
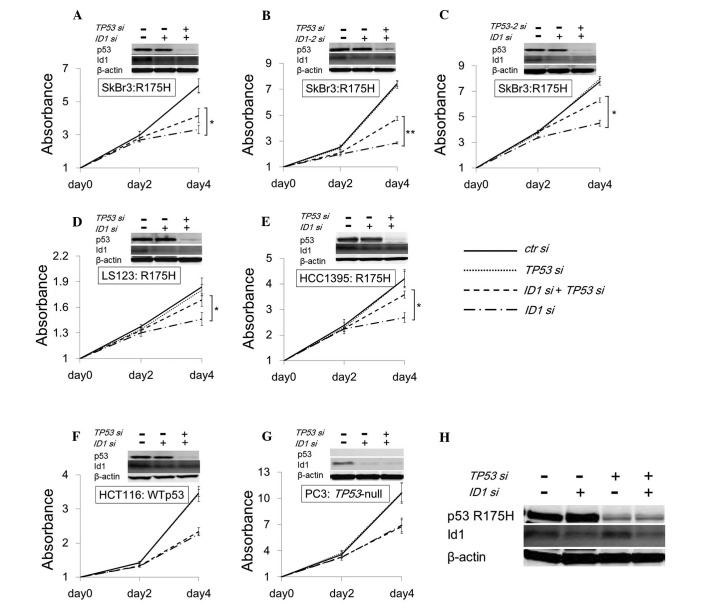
Comparison of cell growth inhibition by *ID1* suppression alone and *ID1*/*TP53* double suppression in cells expressing p53 R175H, wild-type p53, and *TP53*-null cells. (A) *ID1* and *TP53* siRNA were co-transfected into SKBr3 cells to make a final concentration of 100 nM, and cell numbers were measured by performing cell proliferation assays on days 2 and 4. The vertical axis corresponds to the absorbance of the cell proliferation assay. Top of each figure represents expression inhibition of Id1 or p53 by each siRNA. Furthermore, other siRNAs of *ID1* (*ID1*-*2*) (B) and *TP53* (*TP53*-2) (C) targeting different sites were transfected into SKBr3 cells. The same experiments were conducted in LS123 and HCC1395 cells, p53 R175H expressing cell lines (D and E), HCT116 cells expressing wild-type p53 (F), and the *TP53*-null cell line PC3 (G). Values shown are means ± SD (n=3) in (A and E). ^*^p<0.05, ^**^p<0.01, between *ID1* suppression alone and *ID1*/*TP53* double suppression. (H) Western blot analysis of Id1 and p53 R175H. Knockdown level of Id1 was not rescued by *ID1*/*TP53* double suppression in SKBr3 cells.

To determine whether cell growth inhibition is rescued by the suppression of both candidate genes and p53 R175H, siRNAs of the targeting candidate genes and *TP53* were transfected into SKBr3, a p53 R175H expressing cell line. Downregulation of p53 R175H rescued cell growth inhibition caused by *ID1* suppression (Fig. 3A), but not by *GYPC, NUP98, GP6* and *EFNA4* suppression (data not shown). To exclude the off-target effect of siRNA, other siRNA for *ID1* and *TP53* targeting different sites (*ID1-2* and *TP53-2*) were transfected into SKBr3, and we observed reproducible results to the original siRNAs (Fig. 3B and C). Moreover, similar results were observed only in cell lines expressing p53 R175H (LS123, Fig. 3D and HCC1395, Fig. 3E), but not in wt p53 (HCT116, Fig. 3F), and *TP53*-null (PC3, Fig. 3G). The quantity of the Id1 protein in SKBr3 was not altered by p53 R175H suppression (Fig. 3H), same as p53 R175H transient expression. These results support the finding that cell growth inhibition by *ID1* suppression is accelerated by p53 R175H.

### Suppression of ID1 in cell lines expressing another common mutant p53 (R273H)

**Figure 4:**
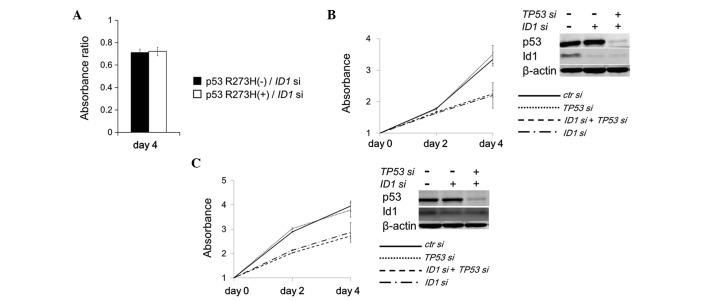
*ID1* suppression in PC3 cells expressing p53 R273H and *ID1*/*TP53* double suppression in cells expressing p53 R273H. (A) The plasmid pCR259-R273H was transfected into PC3 cells for 96 h, and total cell numbers were measured by performing cell proliferation assays. *ID1* siRNA and *TP53* siRNA were co-transfected into HT-29 cells expressing endogenous p53 R273H (B) and SW480 expressing endogenous p53 R273H/R309S double mutant (C). Cell counts were measured by performing cell proliferation assays on days 2 and 4. The vertical axis is same as in Fig. 3. The top right of each figure is the same as that in Fig. 3. Values shown are means ± SD (n=3).

To examine whether cell growth inhibition caused by *ID1* suppression is accelerated specifically by p53 R175H expression, another common p53 mutant (R273H) was expressed in a PC3 cell line (*TP53*-null). Unlike p53 R175H expression, p53 R273H expression did not accelerate the cell growth inhibition caused by *ID1* suppression (Fig. 4A). Furthermore, the cell growth inhibition caused by *ID1* suppression was not restored by simultaneous suppression of *TP53* in HT-29 cells expressing endogenous p53 R273H (Fig. 4B). Similar results were observed in SW480 cells expressing endogenous p53 R273H/P309S double mutants (Fig. 4C). These results indicated that the growth inhibition induced by *ID1* suppression may be accelerated by p53 R175H expression in a specific manner.

### Cell cycle analysis under ID1 suppression and ID1/TP53 double suppression

**Figure 5:**
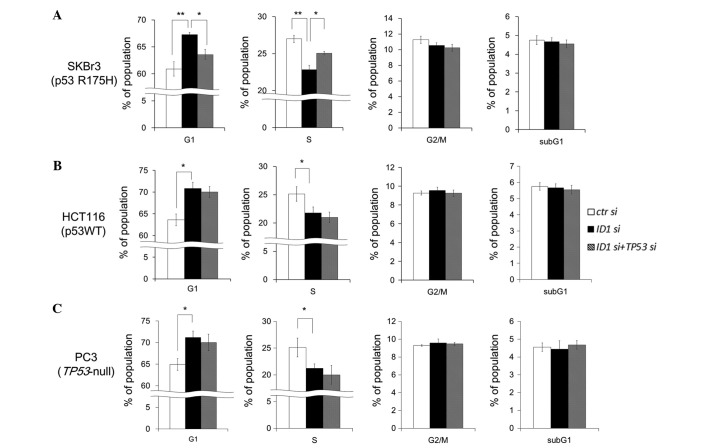
FACS analysis of SKBr3 (A), HCT116 (B) and PC3 (C) cells with *ID1* and *TP53* double suppression. FACS analysis with or without *ID1* siRNA (100 nM) and *TP53* siRNA (100 nM). Values of the sub-G1 fraction are a portion of the total population, and values of G1, S, and G2/M fractions are portions of the population excluding the sub-G1 fraction. Values shown are means ± SD (n=6). ^*^p<0.05, ^**^p<0.01, between negative control and *ID1* suppression or between *ID1* suppression and *ID1*/*TP53* double suppression.

To examine whether *ID1* and/or *TP53* suppression change the proportion of cell cycle phases, FACS analysis was performed in SKBr3 cells. *ID1* suppression did not change the sub-G1 fraction, but significantly decreased the S phase fraction and increased the G1 phase fraction (Fig. 5A). *ID1*/*TP53* double suppression significantly restored the proportion of S phase and G1 phase fractions. These results suggest that p53 R175H potentiates G1 arrest by *ID1* suppression. In HCT116 (wild-type p53) and PC3 (*TP53*-null) cells, *ID1* suppression increased the G1 phase fraction and decreased the S phase fraction. However, unlike in SKBr3 cells, *ID1*/*TP53* double suppression did not restore the proportion of S phase and G1 phase fractions (Fig. 5B and C). These results suggest that *ID1* suppression induces G1 arrest and the arrest is specifically accelerated by p53 R175H expression.

## Discussion

We identified *ID1* as a synthetic sick/lethal gene that caused cell growth inhibition in the presence of p53 R175H. Id1 is a member of the helix-loop-helix protein family expressed in actively proliferating cells and regulates gene transcription by hetero-dimerization with the basic helix-loop-helix (bHLH) transcription factor (25). The homodimer of the bHLH transcription factor activates the differentiation, whereas the heterodimer, composed of Id1 and the bHLH transcription factor, attenuates their ability to bind DNA and consequently inhibits cell differentiation (26). Supporting this finding, stable Id1 expression was found to block B cell maturation (27). Moreover, Id1 can inhibit differentiation of muscle and myeloid cells by associating *in vivo* with E2A proteins (28,29). It has also been reported that Id1 was immunohistochemically expressed in majority of non-small cell lung cancer (NSCLC) samples (30). Furthermore, Id1 protein expression in prostate cancer cells mediated resistance to apoptosis induced by TNFα (31). These lines of evidence also indicate that *ID1* may play an essential role in carcinogenesis.

In the present study, we demonstrated that *ID1* suppression resulted in cell growth inhibition that was independent of *TP53* status. However, cell growth inhibition caused by *ID1* suppression was accelerated specifically by the p53 R175H mutant protein. If the accelerated cell growth inhibition is attributable only to loss-of-function in p53 R175H, this phenomenon should also be observed in *TP53*-null cells and other cells expressing loss-of-function mutations other than p53 R175H. Some p53 mutant proteins acquire additional functions called gain-of-function (32). For example, ectopic expression of p53 R175H resulted in transactivation of genes that are not usually activated by wild-type p53 (33–35). On the basis of these observations, we concluded that the acceleration of cell growth inhibition was likely attributable to gain-of-function of p53 R175H.

To date, synthetic sickness/lethality has been classified into 2 types based on the initial genetic event. The first type is attributable to a loss-of-function mutation in a target gene and the second type is attributable to a gain-of-function or an activated mutation in a target gene. For example, the synthetic lethal interaction between loss-of-function mutations in *BRCA1* and *BRCA2* genes and PARP inhibition (8) are a former type, and gain-of-function mutations in the *KRAS* gene and STK33 inhibition (6) are a latter type. Based on our results, it is clear that the synthetic sick/lethal interaction between p53 R175H expression and *ID1* suppression is of the latter type. However, there is a clear difference between the activated *KRAS*-STK33 interaction and the p53 R175H-Id1 interaction. Gain-of-function in activated K-ras depends on STK33 and is therefore blocked by STK33 suppression. By contrast, the accelerated cell growth inhibition observed here cannot be explained only by blockade of gain-of-function in p53 R175H by *ID1* suppression. By contrast, it is necessary for accelerated growth inhibition by *ID1* suppression. Taken together, these results suggest that the synthetic sickness/lethality of p53 R175H with *ID1* suppression may be through a gain-of-function mechanism that is distinct from the previously identified gain-of-function mechanisms. Since both expression and suppression of p53 R175H had no effect on the amount of Id1 protein, p53 R175H may cooperate with downstream factor(s) that are altered by *ID1* suppression and may promote synthetic sickness/lethality in cooperation with protein(s) downstream of *ID1*.

The precise molecular mechanisms of the synthetic sickness/lethality of *ID1* suppression and p53 R175H expression remain to be elucidated. In conclusion, Id1 and its associated signaling pathway is one of the molecular targets of cancer cells expressing the common p53 mutant R175H.

## Figures and Tables

**Table I:** Sequences of synthesized siRNA for 50 candidate genes.

Gene symbol	siRNA sense sequence	siRNA antisense sequence
Smallest p-value		
*UROS*	CCTCTGTGGAAGCCAGCTTAA	TTAAGCTGGCTTCCACAGAGG
*GYPC*	GCTCAGAACGATTGGAAATAA	TTATTTCCAATCGTTCTGAGC
*PRO1596*	CGATGAATATCTCTGTGAATA	TATTCACAGAGATATTCATCG
*CD69*	GGAGCATTTATAAATGGACAA	TTGTCCATTTATAAATGCTCC
*PDXP*	GGTACCAGTTTAGGTTCCTAA	TTAGGAACCTAAACTGGTACC
*THADA*	CGCTTACAGATGATTCTGAAT	ATTCAGAATCATCTGTAAGCG
*KCNJ10*	GCAGATATCTTGGCCTGGTTA	TAACCAGGCCAAGATATCTGC
*ABCA12*	CCAAATTTCCTCCAACTGCAA	TTGCAGTTGGAGGAAATTTGG
*UBA6*	GCATTGTTACTTGCCTTGAAA	TTTCAAGGCAAGTAACAATGC
*CDC7*	GCACTTTCAGCTCTGTTTATT	AATAAACAGAGCTGAAAGTGC
*ID1*	GGAAATTGCTTTGTATTGTAT	ATACAATACAAAGCAATTTCC
*CTBS*	GGCTCCTTATTATAACTATAA	TTATAGTTATAATAAGGAGCC
*EIF2B3*	CGGAGTGAACTGATTCCATAT	ATATGGAATCAGTTCACTCCG
*UFM1*	GGTAGCAAAGTGTTACAGAAA	TTTCTGTAACACTTTGCTACC
*PTCD1*	CCTCGATGTGTTCAAGGAAAT	ATTTCCTTGAACACATCGAGG
*TPCN2*	GGAGCTCCTGTTCAGGGATAT	ATATCCCTGAACAGGAGCTCC
*NEURL*	GGGTAACAACTTCTCCAGTAT	ATACTGGAGAAGTTGTTACCC
*STEAP4*	GCACTATATTAGGTTAAGTAT	ATACTTAACCTAATATAGTGC
*C19orf40*	GCATATTGTGGCCAATGAGAA	TTCTCATTGGCCACAATATGC
*C19orf38*	CCACCTTGGATGATCACTCAG	CTGAGTGATCATCCAAGGTGG
*C14orf37*	GGAACTCCTTACAAGCACTAA	TTAGTGCTTGTAAGGAGTTCC
Largest fold-change		
*MGC42105*	CCAGCTGACGCCCTTCGAGAA	TTCTCGAAGGGCGTCAGCTGG
*GP6*	GGGCTCCAGACGGATCTCTAA	TTAGAGATCCGTCTGGAGCCC
*BCL2L14*	GCCTGTAGCTTCAAGTTCTAA	TTAGAACTTGAAGCTACAGGC
*HILS1*	GCCAAGTGCCACTGCAATTAA	TTAATTGCAGTGGCACTTGGC
*CLDND2*	GAAGAATGCGTGGAAGAACAA	TTGTTCTTCCACGCATTCTTC
*UMOD*	CCACTGACACCTCAGAAGCAA	TTGCTTCTGAGGTGTCAGTGG
*RHAG*	GGGCATATTCTTTGAGTTATA	TATAACTCAAAGAATATGCCC
*GFPT2*	GCTACGAGTTTGAGTCAGAAA	TTTCTGACTCAAACTCGTAGC
*SATL1*	AAGCATGGATTACTTTCAAAT	ATTTGAAAGTAATCCATGCTT
*RTN4IP1*	GCTGCCAGTGTAAATCCTATA	TATAGGATTTACACTGGCAGC
*DOK7*	CCAAGCGGATTCATCTTTGAA	TTCAAAGATGAATCCGCTTGG
*NUP98*	GGCCAAAGGCTTTACAAACAA	TTGTTTGTAAAGCCTTTGGCC
*PWWP2A*	GCCATGCCGCTCCAAAGTAAT	ATTACTTTGGAGCGGCATGGC
*MEP1A*	GCCTATAAGGCCATCATAGAA	TTCTATGATGGCCTTATAGGC
*CCT6B*	TGGCTGAAGCTCTTGTTACAT	ATGTAACAAGAGCTTCAGCCA
*PDXP*	GGTACCAGTTTAGGTTCCTAA	TTAGGAACCTAAACTGGTACC
*ZNF300*	GCTAATATTAGCTTGTCATAA	TTATGACAAGCTAATATTAGC
*DEFB125*	AGAGGATATAACATTGGATTA	TAATCCAATGTTATATCCTCT
*GJA5*	CGTTGCTCATTAATTCTAGAA	TTCTAGAATTAATGAGCAACG
*EFNA4*	CCATGTTCAATTCTCAGAGAA	TTCTCTGAGAATTGAACATGG
Double entry		
*NMNAT1*	TCATCTGAAGTGTCACGTAAA	TTTACGTGACACTTCAGATGA
*KLHL10*	GCTGAGTACTTCATGAACAAT	ATTGTTCATGAAGTACTCAGC
*LMLN*	CCACAGTGAAACATGAGGTTA	TAACCTCATGTTTCACTGTGG
*FBXO22*	TCCCTCAAATTGAAGGAATAA	TTATTCCTTCAATTTGAGGGA
*ITGB7*	GGACAGTAATCCTCTCTACAA	TTGTAGAGAGGATTACTGTCC
*CPN1*	GGAATGCAAGACTTTAATTAT	ATAATTAAAGTCTTGCATTCC
*COLQ*	GGCTACAATGCTCTTCCTCTT	AAGAGGAAGAGCATTGTAGCC
*AP3B2*	GGATTGCACCTGATGTCTTAA	TTAAGACATCAGGTGCAATCC
*ANXA11*	CGTGGTGAAATGTCTCAAGAA	TTCTTGAGACATTTCACCACG

**Table II:** Top 5 candidate genes for synthetic lethal interaction with R175H on t-test.

Gene symbol	p-value
*GYPC*	0.002446
*NUP98*	0.029324
*GP-6*	0.043416
*EFNA4*	0.055412
*ID1*	0.061552
